# Geo-climatic risk factors for chronic rhinosinusitis in southwest Iran

**DOI:** 10.1371/journal.pone.0288101

**Published:** 2023-07-05

**Authors:** Mohammad Amin Ghatee, Zahra Kanannejad, Koorosh Nikaein, Niloufar Fallah, Gholamabbas Sabz

**Affiliations:** 1 Cellular and Molecular Research Center, Yasuj University of Medical Sciences, Yasuj, Iran; 2 Department of Parasitology, School of Medicine, Yasuj University of Medical Sciences, Yasuj, Iran; 3 Allergy Research Center, Shiraz University of Medical Sciences, Shiraz, Iran; 4 Student Research Committee, Yasuj University of Medical Sciences, Yasuj, Iran; Satyawati College, University of Delhi, INDIA

## Abstract

Chronic rhinosinusitis (CRS) is a prevalent and disabling paranasal sinus disease associated with some environmental factors. In this study, we evaluated the effect of geo-climatic factors on CRS in a region of southwest Iran. The study mapped the residency addresses of 232 patients with CRS who lived in Kohgiluyeh and Boyer-Ahmad province and had undergone sinus surgery from 2014 to 2019. The effects of Mean Annual Humidity (MAH), Mean Annual Rainfall (MAR), Mean Annual Temperature (MAT), maximum MAT (maxMAT), minimum MAT (minMAT), Mean Annual Evaporation (MAE), wind, elevation, slope, and land cover were assessed on the occurrence of CRS using Geographical Information System (GIS). Statistical analysis was performed using univariate and multivariate binary logistic regression. Patients came from 55 points including villages, towns, and cities. In univariate analysis, climatic factors including MAT (OR = 0.537), minMAT (OR = 0.764), maxMAT (OR = 0.63), MAR (OR = 0.994), and MAH (OR = 0.626) were significantly related to CRS occurrence. Elevation (OR = 0.999), slope (OR = 0.9), and urban setting (OR = 24.667) were the significant determinants among geographical factors when analyzed independently. The multivariate analysis found maxMAT (OR = 0.5), MAR (OR = 0.994), elevation (OR = 0.998), and urban (OR = 16.8) as significant factors affecting CRS occurrence. The urban setting is the most critical factor affecting CRS disease. Cold and dry areas and low attitude are the other risk factors for CRS in Kohgiluyeh and Boyer-Ahmad province, southwest Iran.

## Introduction

Chronic rhinosinusitis (CRS) is inflammation of the nasal cavity and paranasal sinuses, often classified based on the duration of symptoms and inflammation: less than one month (acute), between one and three months (sub-acute), and more than three months (chronic) [1]. It is one of the most debilitating diseases, with a prevalence of around 6–15% for acute and 12% for CRS worldwide [2]. CRS management needs frequent outpatient visits and a notable diagnostic and therapeutic measure, which leads to reduced productivity in health services [3].

Climatic change and air pollution are global issues that are largely caused by human activities such as burning fossil fuels, deforestation, and industrialization. It has serious health impacts, as it can cause respiratory tract issues like rhinosinusitis, asthma, and chronic obstructive pulmonary disease (COPD), which can be exacerbated by changes in temperature and humidity [4]. There is a significant association between occupational and environmental risk factors and CRS [5, 6]. Occupational exposure to dust, poisonous gas, noxious inhalant compounds, and environmental factors such as woodstove, indoor tobacco smoke, air pollution, pets, or carpet at home are risk factors for CRS [7, 8]. Airborne diseases, including those caused by bacteria, viruses, and fungi, can also be more easily spread in areas with high levels of air pollution, which can make them particularly problematic in urban areas where air quality is poor and affects CRS [9, 10]. Also, limited ecological studies have been conducted and showed humidity, temperature, and wind speed are climatic risk factors for CRS [7].

Geographical Information System (GIS) has become an essential tool in medical epidemiological studies, monitoring, and control of disease and can be used for public health planning and disease risk forecasting. By GIS, the danger zones of disease are determined, and the relationship between the geographical features and the disease occurrence is examined by preparing thematic graphic maps. Therefore, this technology can facilitate determining the occurrence and distribution of disease and controlling and managing it as quickly as possible [11]. GIS has become an important tool in determining the geo-climatic factors and risk zones for a variety of diseases, including both infectious and non-infectious diseases [12–19].

Finding risk zones of CRS using GIS technology can help researchers reduce the disease’s progression in society and decrease the related complications and costs. There is little information about the effect of environmental factors on CRS by GIS-based approaches. Two studies used this method for ambient exposure characterization, generally by calculating the distance of residence to the source of environmental pollution, such as intensive hog farming, industries, and dust-producing activities [20, 21].

To our knowledge, this is the first comprehensive GIS-based study investigating the relationship between geo-climatic factors and CRS worldwide. The use of GIS can help to identify areas where the environmental conditions are favorable for the development of CRS, as well as areas where the disease is more prevalent. By analyzing factors such as temperature, humidity, evaporation, rainfall, wind speed, elevation, slope, and land cover, we can gain insights into the underlying environmental drivers of the CRS in region of Kohgiluyeh and Boyer-Ahmad province, southwest Iran.

## Materials and methods

### Study area

The study areas included Boyer-Ahmad and Dena counties in Kohgiluyeh and Boyer-Ahmad province with latitudes of 30°9’ and 31°32’N and longitudes of 49°57’and 50°42’, respectively, in southwest Iran. The capitals of Boyer-Ahmad and Dena are Yasuj and Sisakht, respectively. Boyer-Ahmad and Dena counties cover an area of about 65,000 km^2^ and 1,821 km^2^, respectively, and are located in a cold climatic region. These counties include areas in one of the highest altitudes in the central part of the Zagros mountain chain with high annual snow and rainfall. Dena Peak, the ninth highest peak in Iran, with a height of 4,409 meters, is located in this area. The study area has diverse vegetation with nearly 2,000 plant species.

### Data collection

This five-year cross-sectional study included all CRS patients undergoing sinus surgery from 2014 to 2019 in Boyer-Ahmad and Dena counties. Patient data were collected from hospital records. The home addresses of 232 patients were entered in the attributes of the GIS layer of the province’s political divisions. The spatial point layer of the city and villages of the studied counties and the polygonal layer of counties were extracted from whole province layers using ArcMap software. The study was approved by the Ethics Committee of Yasuj University of Medical Sciences (IR.YUMS.REC.1398.143).

### Geo-climatic data

The meteorological data in the study period were acquired from the Kohgiluyeh and Boyer-Ahmad Province Weather Bureau. The data included temperature, humidity, evaporation, and wind speed obtained from six synoptic meteorological stations and rainfall data from 44 rain-gauge stations distributed in the province.

Mean Annual Temperature (MAT), maximum MAT (maxMAT), minimum MAT (minMAT), Mean Annual Rainfall (MAR), Mean Annual Evaporation (MAE), Mean Annual Humidity (MAH), and mean annual wind speed were calculated. The iso-hydral and iso-humid raster layers were generated using the Kriging interpolation method, and the iso-thermal, iso-evaporation, and iso-wind speed layers using the tension-based Spline interpolation model with a resolution grid of 1 × 1 km.

The digital elevation model raster layer and land cover vector layer were retrieved from the Department of Natural Resources in Kohgiluyeh and Boyer-Ahmad province. The slope raster layer was generated based on the Digital Elevation Model (DEM) map by using the spatial analyst tool to calculate the maximum rate of change in the value between each cell and its neighbors.

### Geospatial and statistical analysis

The point layers of cities and villages of the studied counties were extracted with the raster layers, and then the geometric intersections of the obtained layer and land cover vector layer were computed by the identity tool to generate the final layer. Each point represented geo-climatic values of all overlapped raster and vector layers in the final layer. The association between geo-climatic factors and CRS was assessed based on the spatial description of patients in Kohgiluyeh and Boyer-Ahmad province. Residential points data, including CRS reported and non-reported villages and cities, were extracted from the final province villages/cities’ point layers and analyzed using univariate and multivariate logistic regression models. The statistical analyses were performed using SPSS version 21.

## Results

### Geo-climatic distribution of points with CRS

**Fig 1:**
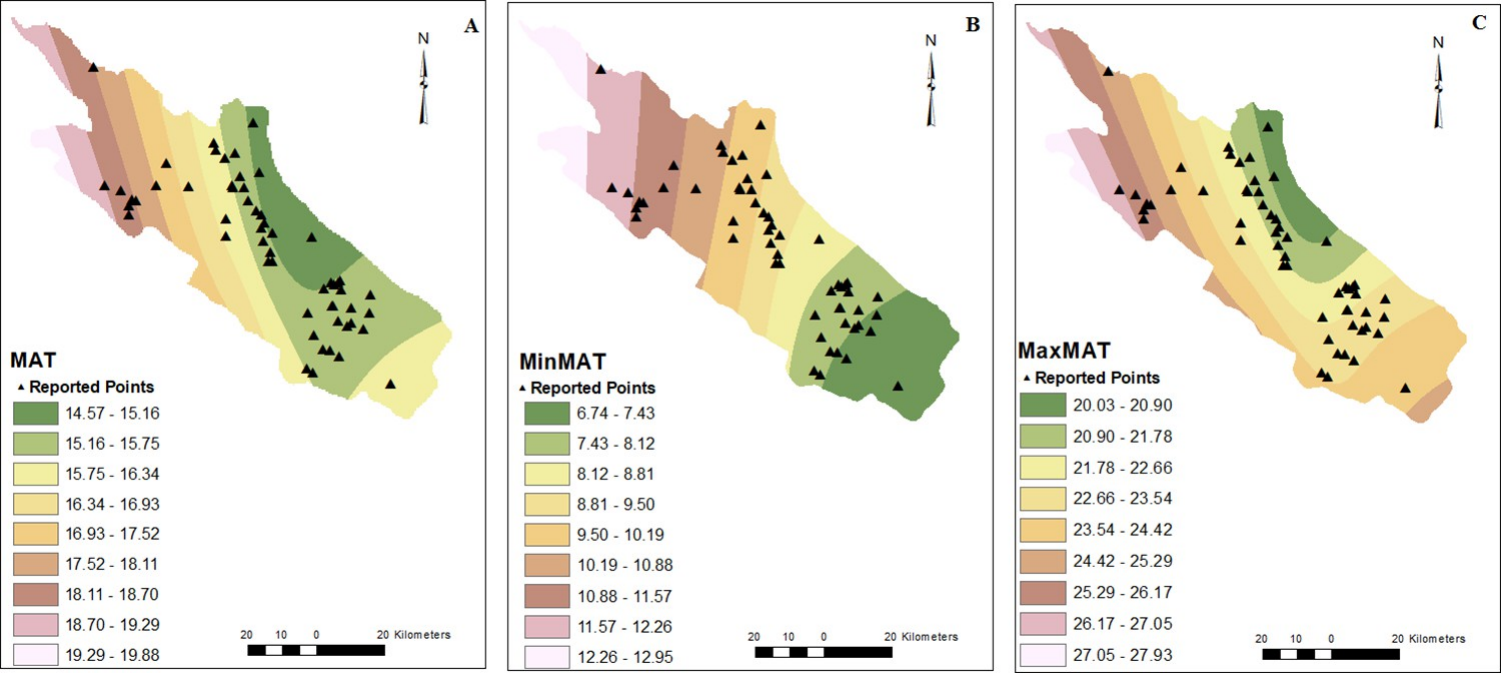
MAT (A), minMAT (B), maxMAT (C) raster models. Points with rhinosinusitis were shown by triangle symbol. Mean annual temperature (MAT), maximum (max), minimum (min).

**Fig 2:**
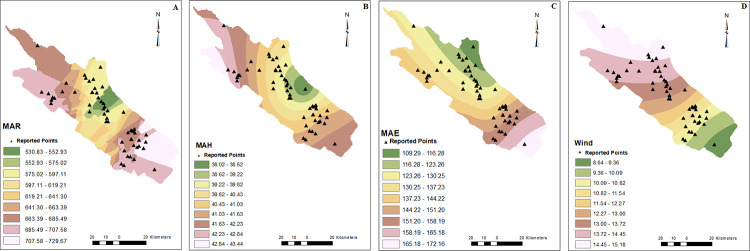
MAR (A), MAH (B), MAE (C), wind speed (D) raster models. Points with rhinosinusitis were shown by triangle symbol. Mean annual rainfall (MAR), mean annual humidity (MAH), mean annual evaporation (MAE).

**Fig 3:**
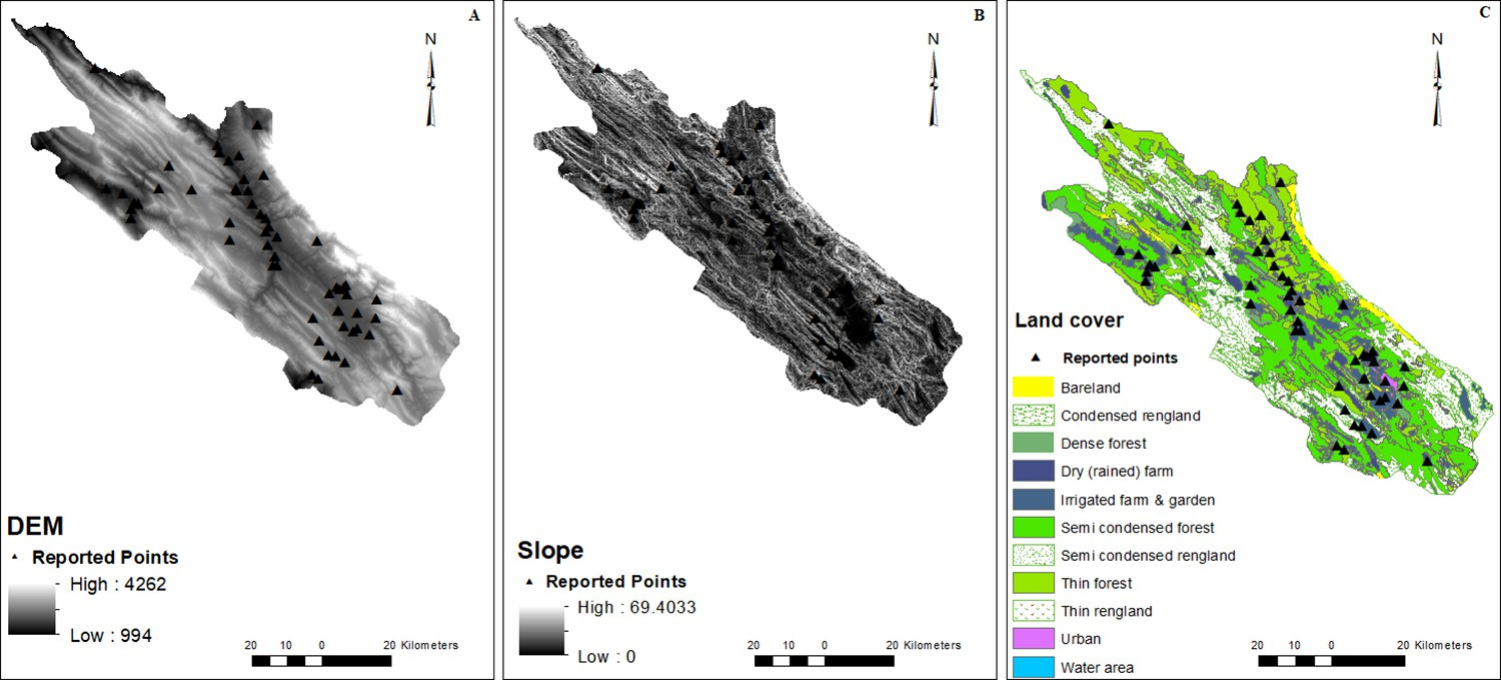
Maps of the geographical factors: DEM (A), slope (B), and land cover maps (C). Points with CRS were shown by triangle symbol. DEM (digital elevation model).

Patients were reported from 55 points in the current study. CRS cases were reported from different areas, but most were from the east and north of the studied area (Figs 1–3).

### Univariate logistic regression

As shown in Table 1, among all climatic factors, MAT, minMAT, maxMAT, MAR, and MAH were detected as significant factors associated with CRS. Each degree increase in MAT (CI = 0.404–0.714, OR = 0.537), minMAT (CI = 0.651–0.895, OR = 0.764), and maxMAT (CI = 0.518–0.764, OR = 0.630) reduced the chance of disease by 46.3%, 23.6%, and 37%, respectively. Besides, MAR (CI = 0.988–0.999, OR = 0.994) and MAH (CI = 0.498–0.788, OR = 0.626) had inverse effects on CRS occurrence. Accordingly, each one-milliliter increase in rainfall and one percentage increase in humidity reduced the chance of disease by 1.6% and 38.4%, respectively. However, MAE and wind speed had no significant effect on the occurrence of CRS.

**Table 1:** Univariate analysis of the effect of climatic factors on CRS.

Variable	P- value	Odd’s ratio	CI
MAT	0.000	0.537	0.404–0.714
min MAT	0.001	0.764	0.651–0.895
max MAT	0.000	0.630	0.518–0.764
MAR	0.025	0.994	0.988–0.999
MAH	0.000	0.626	0.498–0.788
MAE	0.401	0.991	0.969–1.013
Wind speed	0.108	0.887	0.766–1.027

MAT, mean annual temperature;max, maximum;min, minimum;MAR, mean annual rainfall; MAH, mean annual humidity; MAE, mean annual evaporation.

Table 2 shows the effect of geographical parameters on CRS. Urban setting, DEM, and slope were the environmental determinants of CRS. Urban setting increased the odds of CRS by 24.6 folds (CI = 4.534–134.190, OR = 24.667), while DEM (CI = 0.998–0.999, OR = 0.999) and slope (CI = 0.855–0.952, OR = 0.902) decreased the disease occurrence by 0.1% and 9.8%, respectively. Also, there was an inverse trend for semi-condensed forest areas, but it was not significant.

**Table 2:** Univariate analysis of the effect of geographical factors on CRS.

Variable	P- value	Odd’s ratio	CI
Land covers			
Dry (rainfed) farm	0.135	1.665	0.529–1.835
Thin rangeland	0.142	3.171	0.679–14.810
Semi-condensed rangeland	0.107	0.168	0.019–1.474
Condensed rangeland	0.998	0.000	0.000
Sparse forest	0.545	1.403	0.469–4.202
Semi-condensed forest	0.052	0.236	0.055–1.013
Condensed forest	0.991	0.987	0.107–9.063
Urban	0.000	24.667	4.534–134.190
Irrigated farm	0.328	1.638	0.609–4.403
DEM	0.001	0.999	0.998–0.999
Slope	0.000	0.902	0.855–0.952

DEM, Digital elevation model.

### Multivariate logistic regression model

In the multivariate analysis, all significant factors in the univariate regression model were computed by the forward stepwise method of multivariate regression analysis. In this method, only land covers, maxMAT, MAR, and DEM were entered in the last computation step by the model, which appeared as significant variables affecting CRS. As shown in Table 3, among these parameters, the urban setting (CI = 2.696–105.707, OR = 16.88) was the only factor associated with increased CRS occurrence, while there was a decreasing trend for DEM (CI = 0.669–0.999, OR = 0.998), maxMAT (CI = 0.371–0.679, OR = 0.502), and MAR (CI = 0.982–1.020, OR = 0.994). Other geo-climatic factors were not detected as significant variables.

**Table 3:** Multivariate analysis of the effect of geographical factors on CRS.

Variable	P- value	Odd’s ratio	CI
Thin rangeland	0.071	4.379	0.882–21.740
Semi-condensed rangeland	0.353	0.351	0.038–3.230
Condensed rangeland	0.998	0.000	0.000
Sparse forest	0.597	0.717	0.209–2.458
Semi-condensed forest	0.069	0.204	0.045–0.923
Condensed forest	0.517	2.147	0.231–21.608
Urban	0.003	16.881	2.696–105.707
Irrigated farm	0.748	1.185	0.421–3.341
Dry (rainfed) land	0.999	0.000	0.000
max MAT	0.000	0.502	0.371–0.679
MAR	0.010	0.994	0.982–1.020
DEM	0.000	0.998	0.669–0.999

MaxMAT, maximum mean annual temperature; MAR, mean annual rainfall; DEM, digital elevation model.

## Discussion

This study showed that the urban setting, maxMAT, MAR, and DEM had the most effects on CRS in sequence. Besides, MAT, MAH, minMAT, and slope were determining factors when their effects were analyzed independently.

### Geographical factors

Based on the results, among various types of land cover, the urban setting was the most important factor directly affecting CRS occurrence. Our results are consistent with some studies showing a higher prevalence of allergic rhinitis and nasal symptoms among people born and raised in urban areas than among those in rural areas [22–25]. The microbial load and diversity accompanied by farm living in rural areas have been suggested as a reason for the beneficial effects on respiratory diseases [26]. However, some studies failed to identify this protective effect and reported that farming activities and poor hygiene were associated with increased prevalence of rhinitis in such areas [27, 28]. Another possible explanation of the direct association between urbanization and sinus problem is increased air pollution, dust particles, factories, sensitizing chemicals, and waste in urban areas [29–31]. A recent study by Zhang et al. reported long-term exposure to airborne particulate matter ≤2.5 in aerodynamic diameter (PM2.5) as a risk factor for developing CRS in non-allergic patients [32]. In addition, the increasing agglomeration of people in dense urban areas increased the risk of contagious diseases, especially rhino, influenza, and parainfluenza viruses, contributing to disease exacerbation [33–36].

As another influential geographical factor, elevation was inversely associated with CRS in the study area, where more CRS was reported among villages or cities with lower elevation. It may be due to high population density in regions with low elevation in mountainous areas associated with increased air pollutants, nanoparticles, and infectious viral diseases [36]. There was no prior study investigating the effect of altitude on CRS, while some reported the benefits of permanent high-altitude residence for reducing exacerbations [37] and long-term high-altitude residence (>12 weeks) for improving symptoms and lung function [38]. However, individuals whose asthmatic trigger is cold air at lower humidity may be more prone to asthma exacerbations at a high altitude, especially during exercise [39].

We also showed more CRS in areas with a lower slope for the first time. Human populations have more tendencies to areas with lower slopes for ease of movement. So, more populated areas and, accordingly, CRS predisposing factors can be found in lower slope regions.

### Climatic factors

Based on the multivariate logistic study, maxMAT was the most important climatic factor that was inversely related to CRS occurrence. An inverse association also was found for humidity in univariate analysis. While high temperature and humidity are protective factors against CRS, cold and dry climates could worsen its condition. Inhalation of cold and dry air leads to the congestion of elastic tissue of the nose that reduces the heat exchange between the inhaled and exhaled air. Lack of moisture in the air can dry out the sinuses, leading to irritation and thickening mucus. Together, these factors impair mucociliary transport function and result in nasal obstruction, which can be a precursor to CRS [27, 28]. With extra time spent indoors during the colder weather, patients are more prone to indoor allergy attacks, which is one of the potential risk factors for CRS [5]. In addition, cold is the main trigger of viral sinus infection that blocks off the drainage channels of sinuses [40]. Our results contrast with some previous studies that reported a high incidence of CRS at higher temperatures [8]. They believed that the increased temperature and humidity create suitable conditions for fungi growth [8]. However, these studies investigated only fungal CRS, a sinus infection resulting from a fungus, while most surgical CRS cases in this study were allergic or associated with polyps (unpublished data). Furthermore, our study focused on cold mountainous areas where high temperatures can be more suitable for health maintenance.

We detected MAR as a protective factor against CRS by multivariate logistic regression. Rainfall events may eliminate dust particles, air pollutants, and other airborne particles, which are the environmental risk factors for CRS [41, 42]. However, some studies failed to detect a significant association between rain and CRS [41]. In contrast to our findings, increased rainfall was a risk factor for disease progression in patients with fungal rhinosinusitis [43]. In addition, increased MAR was associated with increased humidity, which is another significant factor in our study when analyzed independently. We showed that increased humidity is associated with decreased incidence of CRS. Sinusitis experts agree that adding humidity to the air with a humidifier is generally good for sinus health. Humidity can help nasal congestion by providing more moisture and humidity within the nose. Like rainfall, some studies reported humidity as a negative factor influencing fungal rhinosinusitis [44, 45].

## Conclusions

In short, the urban setting was the most critical risk factor for CRS, and lower altitude and slope were the other geographical factors. This study demonstrated that cold and dry climatic conditions were major meteorological risk factors for CRS development in this region, while higher temperatures, rainfall, and humidity were protective factors. These findings can determine the high-risk areas of CRS in this region of southwest Iran that patients and health professionals should consider. Further research is needed to address the risk factors associated with urban settings like air pollution or socioeconomic factors in a large retrospective study.
